# Supplementary material for: Facilitators and barriers of physical activity, sedentariness and exercise adoption among healthcare professionals in Lagos State, Nigeria – A qualitative review
Source: PLoS One. 2026 Feb 3;21(2):e0342108. doi: 10.1371/journal.pone.0342108 (PMC12867225; doi:10.1371/journal.pone.0342108)
Supplement: S1 File — (PDF) [file pone.0342108.s001.pdf]

Supplementary File

List of Files

Number 1. Qualitative Data Collection Guides

Number 2. Hierarchical coding tree illustrating themes and subthemes derived from thematic analysis guided by the Social Ecological Model.

Number 3. High-Level Codebook for Thematic Analysis (SEM-Guided)

Number 4. Anonymised Excerpts from FGDs, IDIs and KIIs

Number 1: Qualitative Data Collection Guides

A. Focus Group Discussion (FGD) Guide

Target Participants: Healthcare professionals (doctors, nurses, CHEWs/CHOs)

Section 1: Introduction and Consent

- Welcome participants and introduce the moderator.
- Explain the purpose of the study and the focus of the discussion.
- Emphasise voluntary participation, confidentiality, and anonymity.
- Obtain verbal consent for participation and audio recording.
- Encourage open and honest discussion and respect for differing opinions.

Section 2: Participant Introduction and Warm-up

1. Please introduce yourself (profession and years of experience).
2. Warm-up question:
    - Can you briefly describe how physical activity or exercise fits into your daily life, both at work and outside work?

Section 3: Perceptions of Physical Activity and Sedentariness

1. How would you describe the general level of physical activity among healthcare professionals in Lagos State?
2. From your experience, do you think healthcare professionals are generally active or sedentary? Why?
3. Are there specific professions or departments where physical inactivity seems more common?

SECTION 4: DETERMINANTS OF PHYSICAL INACTIVITY AND SEDENTARINESS

1. What factors contribute to physical inactivity or sedentary behaviour among healthcare professionals?
   - ***Probe:*** Work schedules, workload, staffing levels
2. How does your work environment influence your physical activity levels?
3. How do personal factors such as time constraints, family responsibilities, stress, or fatigue affect exercise participation?
4. How do cultural norms or societal expectations influence physical activity and exercise?
5. How does the built environment (e.g., sidewalks, parks, safety, transport) affect your ability to be physically active?

SECTION 5: FACILITATORS AND BARRIERS TO EXERCISE ADOPTION

1. What motivates healthcare professionals to engage in regular exercise?
2. What are the main barriers that prevent exercise adoption?
3. Are there strategies or resources that have helped some colleagues stay active?
4. How can healthcare organisations better support physical activity among staff?
   - ***Probe:*** Facilities, policies, exercise breaks, incentives
5. What role can peer support play in encouraging exercise?

SECTION 6: RECOMMENDATIONS AND FUTURE DIRECTIONS

1. Do you think specific interventions or policies are needed to promote physical activity among healthcare professionals?
2. What role should healthcare professionals play in advocating for change?

3. What are your hopes for the future regarding physical activity among healthcare professionals in Lagos State?

SECTION 7: CLOSING

- Summarise key discussion points.
- Invite final comments.
- Thank participants and reiterate confidentiality.

B. IN-DEPTH INTERVIEW (IDI) GUIDE

Target Participants: Family members of healthcare professionals

SECTION 1: INTRODUCTION AND CONSENT

- Explain study purpose and relevance.
- Assure confidentiality and voluntary participation.
- Obtain verbal consent and permission to record.

SECTION 2: BACKGROUND INFORMATION

1. Relationship to the healthcare professional.
2. How familiar are you with their daily routines and lifestyle?

SECTION 3: PERCEPTIONS OF PHYSICAL ACTIVITY AND SEDENTARINESS

1. How would you describe the physical activity and exercise habits of your relative?
2. Do you think their work influences how active or sedentary they are? How?

SECTION 4: DETERMINANTS OF PHYSICAL ACTIVITY AND EXERCISE

1. What factors make it difficult for them to exercise regularly?
   - ***Probe:*** Time, fatigue, family responsibilities
2. What factors encourage or support them to be physically active?
3. How do family expectations or responsibilities influence exercise behaviour?

SECTION 5: ENVIRONMENTAL AND CULTURAL INFLUENCES

1. How does the home or neighbourhood environment affect opportunities for physical activity?
2. Are there cultural beliefs or norms that influence attitudes toward exercise?

SECTION 6: RECOMMENDATIONS

1. What do you think would help healthcare professionals become more physically active?
2. What role can family members play in supporting exercise?

SECTION 7: CLOSING

- Invite additional comments.
- Thank participant and close interview.

C. KEY INFORMANT INTERVIEW (KII) GUIDE

Target Participants: Policymakers, urban planners, healthcare facility leaders

SECTION 1: INTRODUCTION AND CONSENT

- Explain study objectives and policy relevance.
- Assure confidentiality and voluntary participation.
- Obtain verbal consent and permission to record.

SECTION 2: PROFESSIONAL BACKGROUND

1. Please describe your current role and responsibilities.
2. How does your work relate to public health, urban planning, or infrastructure?
3. To what extent does your role influence policies affecting physical activity?

SECTION 3: PERCEPTIONS OF PHYSICAL ACTIVITY AMONG HEALTHCARE PROFESSIONALS

1. How would you describe physical activity levels among healthcare professionals in Lagos State?
2. What factors contribute to physical inactivity and sedentariness in this group?
    - ***Probe:*** Workload, understaffing, urban context

SECTION 4: POLICY ATTENTION AND PRIORITIES

1. Has physical inactivity among healthcare professionals received adequate policy attention? Why or why not?
2. Are there existing policies aimed at promoting physical activity?
3. What competing priorities affect policy focus on physical activity?

SECTION 5: URBAN ENVIRONMENT AND INFRASTRUCTURE

1. How does Lagos' urban environment influence opportunities for physical activity?
2. What environmental barriers limit active living?
3. How well do urban planning policies incorporate physical activity–friendly spaces?

SECTION 6: TRANSPORTATION AND MOBILITY

1. How does transportation infrastructure affect physical activity?
2. Are there initiatives promoting active transport?
3. What challenges limit their effectiveness?

SECTION 7: POLICY GAPS, CHALLENGES, AND SOLUTIONS

1. What challenges exist in implementing physical activity–promoting policies?
2. What policy changes would most improve physical activity among healthcare professionals?
3. How can collaboration between sectors be strengthened?

SECTION 8: ADVOCACY AND THE WAY FORWARD

1. What role can healthcare professionals play in policy advocacy?

2. What is the most urgent action government should take?

SECTION 9: CLOSING

- Invite additional insights.
- Thank participant and close interview.

S1 TABLE. FOCUS GROUP DISCUSSION (FGD) GUIDE AND THEMATIC ALIGNMENT

Section	Question	Indicative Theme(s)	Primary Code(s)	SEM Level
Introduction	Warm-up: Personal experiences with physical activity	Personal experience with exercise	Past exercise habits; Current activity patterns	Individual
Perceptions	Prevalence of physical inactivity among HCPs	Perceived prevalence of inactivity	Physical inactivity prevalence	Individual
Perceptions	Differences across professions/departments	Occupational differences	Specialty-related activity patterns	Organisational
Determinants	Factors contributing to inactivity	Determinants of inactivity	Workload; Time constraints; Fatigue	Individual
Determinants	Work environment influence	Workplace determinants	Shift work; Staffing shortages	Organisational
Determinants	Personal responsibilities and stress	Competing priorities	Family responsibilities; Stress	Interpersonal
Determinants	Cultural norms and expectations	Cultural influences	Social perceptions of exercise	Community
Determinants	Built environment challenges	Environmental barriers	Safety; Sidewalks; Parks	Community
Facilitators	Motivators for exercise	Exercise motivators	Health benefits; Weight control	Individual
Facilitators	Barriers to exercise adoption	Barriers to exercise	Time; Motivation; Cost	Individual

Facilitators	Organisational support mechanisms	Organisational facilitators	Workplace wellness; Facilities	Organisational
Facilitators	Role of peer support	Social support	Peer encouragement	Interpersonal
Recommendations	Need for interventions/policies	Policy needs	Workplace PA policies	Policy
Future	Hopes for the future	Future outlook	Advocacy; Behaviour change	Policy

S2 Table. In-Depth Interview (IDI) Guide and Thematic Alignment

Section	Question	Indicative Theme(s)	Primary Code(s)	SEM Level
Background	Relationship and familiarity with HCP routines	Family context	Household routines	Interpersonal
Perceptions	Description of HCP activity levels	Observed activity patterns	Sedentariness	Individual
Determinants	Work influence on activity	Occupational strain	Long hours; Fatigue	Organisational
Determinants	Barriers to exercise	Competing demands	Family duties; Time	Interpersonal
Facilitators	Encouraging factors	Support mechanisms	Family encouragement	Interpersonal
Environment	Home/neighbourhood influence	Environmental constraints	Safety; Space	Community
Culture	Cultural beliefs about exercise	Cultural norms	Attitudes to fitness	Community
Recommendations	Strategies to increase activity	Support strategies	Family-based support	Interpersonal

S3 Table. Key Informant Interview (KII) Guide and Thematic Alignment

Section	Question	Indicative Theme(s)	Primary Code(s)	SEM Level
Background	Role and policy responsibilities	Policy context	Governance roles	Policy
Perceptions	Activity levels among HCPs	Perceived inactivity	Workforce inactivity	Individual

Determinants	Causes of inactivity	Structural determinants	Overwork; Understaffing	Organisational
Policy Attention	Policy prioritisation	Policy neglect	Competing priorities	Policy
Policy Attention	Existing PA policies	Policy landscape	Absence of HCP-specific PA policy	Policy
Urban Environment	Effect of urban design	Built environment	Walkability; Safety	Community
Infrastructure	Planning for PA spaces	Planning gaps	Parks; Recreational facilities	Community
Transport	Mobility systems and PA	Active transport	BRT; Rail; Walkability	Community
Challenges	Barriers to implementation	Implementation challenges	Funding; Enforcement	Policy
Solutions	Recommended policy actions	Policy solutions	Workplace PA policies; Urban design	Policy
Advocacy	Role of HCPs	Advocacy potential	Evidence-based advocacy	Interpersonal
Way Forward	Urgent government actions	Strategic priorities	Multisectoral collaboration	Policy

S4 TABLE: HIERARCHICAL CODING TREE ILLUSTRATING THEMES AND SUBTHEMES DERIVED FROM THEMATIC ANALYSIS GUIDED BY THE SOCIAL ECOLOGICAL MODEL.

SEM Level	Theme	Subthemes / Codes (Illustrative)
**Individual**	**Personal Decision and Agency**	Personal choice; individual decision-making; habits; motivation; laziness; personal responsibility
	**Importance of Physical Activity and Exercise**	Health benefits; preventive benefits; weight maintenance; aging gracefully; personal motivation
	**Individual Barriers to Exercise**	Time constraints; stress; emotional exhaustion; low motivation; competing priorities
	**Individual Motivators for Exercise**	Tangible results; health improvement; flexibility; time availability
**Interpersonal**	**Family and Social Influence**	Family responsibilities; family encouragement; influence of family members
	**Peer Influence and Support**	Peer support; social encouragement; leading by example; professional norms

**Organisational (Workplace)**	**Work Environment and Specialty Influence**	Work schedules; workload; prolonged sitting; specialty impact; movement-intensive vs. sedentary specialties
	**Accessibility within the Workplace**	Stair vs. elevator access; proximity of amenities; layout of facilities; time availability
	**Lack of Organisational Support**	Absence of structured exercise programs; limited prioritisation of staff physical activity
	**Role of Organisations in Promoting Exercise**	Workplace policies; leadership involvement; awareness creation; non-monetary incentives; exercise breaks
	**Facilities and Infrastructure (Workplace)**	Gym facilities; sports courts; well-lit outdoor areas; subsidised or free access
**Community / Built Environment**	**Built Environment and Infrastructure**	Parks; sidewalks; walkability; transportation systems; accessibility
	**Safety and Security**	Insecurity; poorly lit spaces; unsafe neighbourhoods
	**Cultural Norms and Expectations**	Attitudes towards physical activity; body image norms; societal expectations; transportation choices
**Policy / Societal**	**Environmental and Policy Influence on Physical Activity**	Organisational policies; urban planning; transport policy; environmental design
	**Messaging and Marketing**	Health messaging; attractiveness; social benefits; preventive framing
	**Strategies to Promote Physical Activity**	Policy implementation; facility provision; incentives; awareness campaigns
	**Role of Healthcare Professionals**	Advocacy; role modelling; pushing for institutional and policy change
	**Hope for the Future and Change**	Growing awareness; potential for change; need for proactive efforts
	**Dissemination and Use of Research**	Utilisation of research findings; advocacy for policy change

S5 TABLE: HIGH-LEVEL CODEBOOK FOR THEMATIC ANALYSIS (SEM-GUIDED)

Code Name	Brief Definition	SEM Level
Personal Choice	Individual decisions and habits related to engaging in physical activity or exercise	Individual
Personal Motivation	Internal drive or willingness to engage in physical activity	Individual

Health Benefits	Perceived physical and mental health benefits of exercise	Individual
Preventive Health	Use of physical activity to prevent illness or maintain long-term health	Individual
Weight Maintenance	Exercise for weight control or body management	Individual
Aging Gracefully	Exercise motivated by desire for healthy aging	Individual
Time Constraints (Individual)	Lack of personal time due to competing priorities	Individual
Stress and Emotional Exhaustion	Psychological or emotional fatigue limiting physical activity	Individual
Family Responsibilities	Household or caregiving roles affecting time for exercise	Interpersonal
Family Support	Encouragement or influence from family members	Interpersonal
Peer Support	Support or encouragement from colleagues or peers	Interpersonal
Social Norms (Peer)	Workplace or professional norms influencing activity behaviour	Interpersonal
Work Schedules	Shift work, long hours, or inflexible schedules affecting activity	Organisational
Specialty Impact	Influence of medical specialty on physical activity levels	Organisational
Workplace Accessibility	Physical layout affecting ease of movement (e.g., stairs, elevators)	Organisational
Organisational Support	Degree to which the organisation prioritises staff physical activity	Organisational
Workplace Policies	Formal or informal policies related to exercise or wellness	Organisational
Leadership Involvement	Role of leaders in promoting physical activity	Organisational
Workplace Facilities	Availability of gyms, sports courts, or exercise spaces	Organisational
Built Environment	Presence of infrastructure supporting physical activity (parks, sidewalks)	Community
Transportation Systems	Transport options influencing active or sedentary behaviour	Community
Safety and Security	Perceived or actual safety concerns limiting outdoor activity	Community
Cultural Norms	Cultural beliefs and expectations about physical activity and lifestyle	Community
Body Image Norms	Social perceptions related to appearance and exercise	Community
Policy Environment	Broader policy context influencing physical activity	Policy
Urban Planning	Design of cities and spaces affecting movement	Policy
Health Promotion Messaging	Communication strategies promoting physical activity	Policy
Incentives	Non-monetary or policy-based incentives for exercise	Policy
Advocacy	Actions taken to promote physical activity at organisational or policy levels	Policy
Role Modelling	Healthcare professionals demonstrating healthy behaviours	Policy
Research Utilisation	Use of research findings to inform practice or policy	Policy

Hope for Change	Perceived potential for improvement in physical activity promotion	Policy

ANONYMISED EXCERPTS FROM FGDS, IDIS AND KIIS

Note: All identifying information has been removed. Participants are identified using generic labels indicating professional group and focus group type. Full transcripts are not publicly available due to ethical and confidentiality considerations.

S6 TABLE: SOCIO-DEMOGRAPHIC DATA OF FOCUS GROUP DISCUSSION (FGD) PARTICIPANTS

FGD Nos.	Participants	Sex	Age	Exercise adoption	Designation
**FGD 1**	Speaker A	Female	41	Adopter	Senior Registrar
	Speaker B	Female	35	Adopter	Senior Registrar
	Speaker C	Male	31	Adopter	Junior Registrar
	Speaker D	Female	39	Adopter	Senior Registrar
	Speaker E	Male	40	Adopter	Senior Registrar
	Speaker F	Female	38	Adopter	Junior Registrar
**FGD 2**	Speaker A	Female	49	Non-Adopter	Consultant
	Speaker B	Female	45	Non-Adopter	Consultant
	Speaker C	Female	52	Non-Adopter	Consultant
	Speaker D	Male	47	Non-Adopter	Consultant
	Speaker E	Female	50	Non-Adopter	Consultant
	Speaker F	Male	42	Non-Adopter	Consultant
	Speaker G	Female	46	Non-Adopter	Consultant
	Speaker H	Female	45	Non-Adopter	Consultant
	Speaker I	Female	42	Non-Adopter	Consultant

**FGD 3**	Speaker A	Female	53	Non-Adopter	Nurse
	Speaker B	Female	35	Non-Adopter	Nurse
	Speaker C	Female	25	Non-Adopter	Nurse
	Speaker D	Female	32	Non-Adopter	Nurse
	Speaker E	Female	38	Non-Adopter	Nurse
	Speaker F	Female	55	Non-Adopter	Nurse
**FGD 4**	Speaker A	Female	40	Adopter	Nurse
	Speaker B	Female	38	Adopter	Nurse
	Speaker C	Female	31	Adopter	Nurse
	Speaker D	Female	35	Adopter	Nurse
	Speaker E	Female	39	Adopter	Nurse
	Speaker F	Female	42	Adopter	Nurse
**FGD 5**	Speaker A	Female	53	Adopter	CHO*
	Speaker B	Female	29	Adopter	CHO
	Speaker C	Female	22	Adopter	CHEW**
	Speaker D	Female	30	Adopter	CHO
	Speaker E	Female	48	Adopter	CHEW
	Speaker F	Male	43	Adopter	CHO
**FGD 6**	Speaker A	Female	26	Non-adopter	CHO
	Speaker B	Female	29	Non-adopter	CHEW
	Speaker C	Female	27	Non-adopter	CHEW
	Speaker D	Female	35	Non-adopter	CHEW
	Speaker E	Female	32	Non-adopter	CHO
	Speaker F	Female	30	Non-adopter	CHO

***Community Health Officer **Community Health Extension Worker; Mean age 38.7(sd9.2); Females 82.7%, Males 17.3%**

Focus Group Discussions

Theme 1:

Personal Experiences with Physical Activity, Sedentariness and Exercise Adoption Among Healthcare Professionals

Participants reflected on their past and present experiences with physical activity and exercise, revealing numerous challenges that hindered their consistency in maintaining a regular exercise routine. Across various focus groups, healthcare professionals (HCPs) shared their struggles with integrating exercise into their busy lives, highlighting common barriers such as demanding work schedules, lack of conducive environments, and competing priorities.

FGD1 Speaker A, Female, 41 years, lamented how life complexities led to exercise becoming a lesser priority over time: *"As I grew older, life grew more complex. One thing or the other just gets in the way and so I just leave exercise to stretches majorly."* Similarly, FGD1 Speaker B, Female 35 years, highlighted her inconsistent exercise regimen, attributing it to various barriers such as work demands and lack of conducive environments: *"So sometimes I do it, sometimes I do not. What I have found to maybe help me or that used to help me exercise in the past, even as an adult, was facilities."*

Participants described varying levels of physical activity in their daily lives. FGD2 Speaker C, Female 52 years, made a conscious effort to remain active despite professional constraints, emphasizing regular breaks and movement during consulting sessions: *"I consciously try to not sit down for prolonged periods without standing up for a five-minute break… I try to encourage others to do the same thing."* FGD2 Speaker D, Male 47 years, reflected on a decrease in physical activity over the years despite a background in sports, indicating a need for more intentional efforts: *"As the years go by, the level of exercising has reduced… Maybe there's a need for more intentionality in ensuring that exercise goes on."*

FGD2 Speaker H, Female 45 years, expressed a similar sentiment, acknowledging restlessness but difficulty in sustaining exercise routines: *"Exercise is a no-no for me… I tried several times, then stopped."* FGD3 Speaker A, Female 53 years, articulated the challenges faced in finding time and energy for exercise amidst demanding work schedules and daily responsibilities: *"By the time you get home, the last thing on your mind is to exercise... there is absolutely no time even if you desire to do the exercise."* FGD3 Speaker F, Female 55 years, noted that nurses are physically active during work hours due to the nature of their jobs, but structured exercise is difficult to factor into a busy day: *"Not during work time, but during leave... I do only what I can."*

Participants observed that colleagues engage in physical activities during work but often neglect structured exercise routines due to time constraints. FGD3 Speaker B, Female 35 years, expressed skepticism about the exercise habits of colleagues, attributing physical fitness to genetics rather than deliberate exercise efforts: *"What I observe in our colleagues is that they are doing physical activities because there is no time for exercise."* FGD3 Speaker F, Female 55 years, recounted an instance of a colleague who registered at a gym but couldn't maintain a regular exercise

routine due to conflicting work schedules and fatigue: *"There's one of our colleagues that was engaged in the exercise before, but she couldn't keep up. I don't think she goes again."*

Participants described how they integrate physical activity into their workdays, such as moving around their healthcare facilities to attend to patients, conducting inspections, or performing cleaning duties. FGD4 Speaker C, Female 31 years, noted: *"...at work, most times I check different departments... Then in exercise, sometimes I do... I have my videos... And I will do the exercise."*

Many participants mentioned walking or engaging in physical activity during their commute to and from work, such as walking from the bus stop or trekking to their workplaces. FGD4 Speaker E, Female 39 years, shared: *"When I'm coming to work, most times I do brisk walking... I stand up from my seat and go to the family planning room... When I'm going back home, I brisk walk but it's not every day."*

Some participants described engaging in exercise routines at home, such as jogging, following exercise videos, or climbing stairs to maintain their physical fitness. FGD4 Speaker D, Female 35 years, noted: *"Then at home, I do weekend jogging... I started with 1,500 steps... I lost 14 kg."*

Participants demonstrated intentional efforts to stay active, such as choosing to walk instead of driving. FGD4 Speaker F, Female 42 years, shared: *"I actually used to drive... but I decided to park it and take a walk from my house to the bus stop... because I was actually adding weight..."* They noted that their colleagues engage in multitasking and are often on their feet, moving around to fulfill various responsibilities within the healthcare setting. FGD4 Speaker B, Female 35 years, commented: *"Most of us at my facility, most of our colleagues, all of us, we do the work together... When you finish clerking a patient, writing cards for them, you move around to get whatever you need."*

Other participants observed that colleagues may prefer to engage in physical activity at home due to personal preferences or limitations within the workplace environment. FGD4 Speaker D, Female 35 years, stated: *"Some people also prefer to do more of physical activities at home than at work... because of exhaustion, because of sweating."*

They additionally highlighted that staff shortages necessitate active participation from all team members, leading to increased physical activity and multitasking to ensure workflow efficiency. FGD4 Speaker F, Female 42 years, explained: *"As others have actually said, we are short-staffed... everybody is on his or her toes. You must get up to do one thing or the other for the work to be done."* The dialogue revealed diverse perspectives on personal physical activity levels, ranging from regular exercise routines to spontaneous physical activities and sedentary behavior. Some participants reported engaging in planned exercise routines, such as jogging or attending fitness sessions on weekends. FGD5 Speaker D, Female 30 years, shared: *"I do jog on weekends."* FGD5 Speaker F, Male 43 years, noted: *"...on a daily basis...I do a lot of exercise, but mostly they are not planned."*

Many participants identified routine daily activities, such as commuting to work, household chores, and walking, as forms of physical activity. These activities were integrated into their daily lives, contributing to overall movement

and energy expenditure. FGD5 Speaker C, Female 27 years, explained: *"...from bus stop to working place or from my bus stop to my house. So that is physical activities."* FGD5 Speaker A, Female 26 years, added: *"...walking from one place to another is a physical activity."* Some participants described engaging in unplanned physical activities, such as walking long distances or performing household tasks, as part of their daily routine. FGD5 Speaker F, Male 43 years, stated: *"...on a daily basis...I can walk. I can walk from here to Mushin."* FGD5 Speaker E, Female 48 years, mentioned: *"...sometimes at home I do a little bit of those stretching stuff."*

Participants acknowledged periods of sedentary behavior during work hours, such as sitting at desks or attending to patients. These sedentary activities contrasted with their active engagement in physical activities outside of work. FGD5 Speaker C, Female 22 years, noted: *"...when you're at work, physically, you go from one place to another."* FGD5 Speaker B, Female 29 years, added: *"..but sometimes when you are attending to your patients you are sedentary..."*

They also shared their observations and perceptions regarding the physical activity levels and sedentary behavior of their colleagues within the healthcare profession. FGD5 Speaker C, Female 22 years, highlighted that most colleagues are physically active, often engaged in various activities both at work and at home: *"Most people I know, my colleagues, they are physically active... most times we are physically active."* FGD5 Speaker D, Female 30 years, emphasized the inherent physical activity involved in certain job roles within healthcare settings, particularly for the lower cadre: *"Let's say most workers are physically active because... you are just moving around...you have to be agile."*

While physical activity was recognized, sedentary behavior was also acknowledged, particularly during periods of rest or relaxation. Participants noted that colleagues may engage in sedentary activities, such as watching TV, after work hours. FGD5 Speaker C, Female 22 years, observed: *"Not everyone really has the time to devote for exercise... maybe just sitting down, relaxed, watching TV..."*

One participant emphasized the demanding nature of healthcare work, stating: *"Majority of us, you know, being that we are short-staffed, most of the health workers, you know, are always physically active. We work all through." (FGD6 Speaker A, Female 26 years)* Some expressed their belief regarding physical inactivity among healthcare professionals in Lagos State. FGD1 Speaker B, F35 highlighted the discrepancy between knowledge and action, noting that while healthcare professionals are aware of the importance of exercise, motivation often falls short, leading to poor adherence to physical activity routines. This feeling was also shared by others. *"We know what we should do but we do not do it. It's not important enough for us to create time out of all the many things that are struggling for our attention."* (*FGD1 Speaker B, Female 35 years)*

Theme 2:

Barriers to Physical Activity, Sedentariness and Exercise Adoption Among Healthcare Professionals

Discussions regarding the factors influencing physical activity, exercise, and sedentary behavior among healthcare professionals revealed a complex interplay between specialty, cadre, job demands, and personal choices. Participants

highlighted specialty-specific obstacles contributing to physical inactivity, such as the sedentary nature of certain medical fields. Disparities in activity levels were noted based on professional rank, with higher-ranked staff, such as consultants, often engaging in less physical activity compared to junior staff like house officers and nurses, who are more physically active due to their duties. Personal agency was also recognized as crucial in prioritizing physical activity despite healthcare profession demands.

Barriers hindering exercise adoption included time constraints and lack of motivation. FGD1 Speaker E, Male 40 years highlighted, *"Time is a barrier because I'm someone who is involved in a lot outside of my regular job."* Stress and mental well-being also played roles in exercise behavior, with FGD1 Speaker F, Female 38 years noting, *"When I become stressed, especially emotionally, and I'm kind of down, I don't really... I lose that motivation."* Environmental barriers, such as the lack of conducive spaces for physical activity, were emphasized by FGD1 Speaker B, Female 35 years: *"So environment is a big factor. It's my major factor."* FGD1 Speaker B, Female 35 years emphasized that while a conducive environment may facilitate exercise, the motivation to exercise lies within oneself: *"So it's not about not having a conducive environment, just the willingness, it's about making the decision to do it."* Others highlighted the need for intrinsic motivation to overcome external barriers and prioritize physical activity.

Individual-level factors, such as family responsibilities, and stress, were significant impediments to exercise adoption. Participants acknowledged the competing demands of work and family obligations, often leaving little time or energy for structured exercise. FGD1 Speaker F, Female 38 years noted, *"Irrespective of all the hindrances, one can still make a decision and maybe help one another to adhere to it,"* underscoring the importance of intrinsic motivation and social support. Challenges in adopting regular exercise routines were also noted. FGD2 Speaker B, Female 45 years cited work demands as a barrier, noting sedentary behavior during consultations: *"I think I am more of a sedentary person because of the kind of work I do… I can be seated for 4 hours consulting without getting up."* FGD2 Speaker F, Male 42 years admitted to a lack of interest in exercise and emphasized work demands as a form of physical activity: *"The exercise, I don't even think I'm thinking about it at all… Work takes me around, I move around quite some."*

Participants also highlighted professional roles' impact on physical activity, with FGD2 Speaker D, Male 47 years attributing the sedentary nature of their work as a laboratory physician to long hours reviewing results: *"My specialty has largely influenced my physical activity."* Environmental factors were significant barriers to exercise adoption. FGD2 Speaker C, Female 52 years emphasized the lack of suitable facilities near their residence: *"Not having the facilities near my home or within my home environment is another issue… Saying that one wants to pay and go to a gym to do exercise is tough."* Time constraints emerged as a common barrier, with participants expressing challenges in finding time for exercise amidst busy work and family schedules. Participants highlighted personal responsibilities such as cooking, childcare, and family obligations as significant barriers to exercise engagement. FGD3 Speaker D, Female 32 years emphasized, *"You get home after much work; you still go and cook... that alone is another exercise."* Work-related stress, compounded by domestic responsibilities, was cited as a

significant barrier to exercise engagement: *"Stress, maybe all the stress from work... And at home again, the same stress."*

Procrastination also emerged as a barrier, with individuals struggling to commit to regular exercise routines. FGD4 Speaker C, Female 31 years noted, *"For me, time wasn't my factor, but it was about making the decision... So, it's about deciding, making a decision to do it and not procrastinating."* The absence of a workout partner or someone to provide motivation was also cited as a significant barrier: *"It's just a lack of motivation or someone to encourage you to do it... It helps when you have people that can encourage you." (FGD4 Speaker A, Female 40 years and FGD4 Speaker B, Female 38 years).*

Theme 3:

Facilitators of Physical Activity and Exercise Adoption among Healthcare Professionals

Despite these barriers, some participants highlighted the inherent physical activity in their job roles, which contributes to their overall fitness level. FGD5 Speaker F, Male 43 years emphasized, *"As a health worker, I'm always on the move. Also, I exercise because I was trying to lose weight. I don't want to gain weight."* Participants also highlighted personal motivations such as weight management and health maintenance as strong facilitators for engaging in exercise.

FGD6 Speaker A, Female 26 years emphasized the importance of organizational support and structured exercise programs as facilitators, suggesting, *"If they can structure an exercise plan for all the staff, it will help us exercise more. If they can organize exercise equipment for us, we will use them."* Role models and supportive environments were also noted as motivators for exercise behavior. FGD6 Speaker E, Female 32 years stated, *"When you look at someone that exercises, you have this feeling that this person is living right. We need someone that can show us that yes, this is what he or she has been doing, and we can do it too."*

FGD1 Speaker E, Male 40 years emphasized the importance of personal motivation and awareness of the health benefits of exercise: *"Irrespective of all that, I am personally motivated by knowing the health benefits of exercise."* FGD1 Speaker D, Female 39 years echoed this sentiment, highlighting health consequences as a primary motivator for engaging in physical activity. Additionally, participants emphasized the significance of time and a safe environment as facilitators of exercise adoption. FGD1 Speaker E, Male 40 years noted the challenge of time constraints, while FGD1 Speaker A, Female 41 years emphasized the importance of a safe environment for encouraging physical activity: *"If I live in a safe environment, I will take regular walks."*

The discussion emphasized the significance of leadership and peer support in fostering a culture of physical activity. Participants shared success stories where teaming up with peers or hiring fitness instructors facilitated exercise adherence. FGD1 Speaker F, Female 38 years recounted, *"My success story is when I teamed up with my sister and hired a gym instructor. Even when he stopped, we were able to continue."* Participants recognized the health benefits of exercise, such as alleviating knee pain, improving mobility, and enhancing overall well-being, as motivating factors for engaging in physical activity. *"...one of the reasons why I decided to personally start*

exercising was that I started having cramps on my knees and some body parts... so it was health-related." (FGD4 Speaker B, Female 38 years) Participants expressed a desire to live long and recognized exercise as a means to maintain fitness and health as they age. *"...everybody wants to live long... exercise will help us keep fit... Almost every food we eat is carbohydrates... In order for us to live long, we must learn how to burn those fats. And the only way we can burn fat is through exercise." (FGD4 Speaker C, Female 31 years)*

Theme 4:

Factors Contributing to Sedentary Behaviour Among Healthcare Professionals

In examining the factors that contribute to sedentary behavior among healthcare participants identified various facilitators that make it easy for them to engage in sedentary activities.

Participants highlighted tiredness as a significant factor that promotes sedentary behavior. The exhaustion from daily activities often leads individuals to seek relaxation by sitting and resting. *"When there is light, they are fond of watching movies. They can be there for 5 hours watching. They are comfortable sitting when there is light, watching all this Africa Magic." (FGD5 Speaker F, Male 43 years) "You always have one or two things to do...let me sit down and just relax my brain a little...before I will now start with every other thing I want to do." (FGD5 Speaker C, Female 22 years)*

Access to entertainment sources such as television, Korean dramas, and social media were identified as key contributors to sedentariness. Participants noted spending extended periods sitting and engaging with screens for leisure. *"People that are into social media, you can sit down and be on Instagram scrolling through for 2 hours...you're being sedentary." (FGD5 Speaker A, Female 53 years) "Some people, they can press phone...when you are in a bus and there is traffic...You can press phone till you get to your bus stop." (FGD5 Speaker B, Female 29 years)* The demanding environment of Lagos, characterized by heavy traffic and long commuting times, further exacerbates sedentariness, they claimed. Participants emphasized how traffic conditions force them into prolonged periods of sitting during their commute. *"Most people are not sedentary because they really want to be...they don't have that luxury of time...Lagos environment contributes to sedentary behaviour." (FGD5 Speaker C, Female 22 years)*

FGD6 Speaker E, Female 32 years highlighted the impact of depression and poverty on sedentary behavior, stating, "*For instance, if you are depressed, some people depression can cause it. Yes, poverty too can cause it.*" They explained that when one is depressed, they would not want to be active. On poverty, they believed that when people are broke, they would spend most of their time indoors to save money. They highlighted the role of economic stability in facilitating physical activity engagement. FGD6 Speaker D, Female 35 years also echoed the influence of financial constraints on sedentary behavior, emphasizing, "*So if I was broke and I don't have money, I would stay at home and say let me just lie down.*" FGD6 Speaker C, Female 27 years took this further and pointed out the impact of unemployment on sedentariness, stating, "*Unemployment can also cause it... You will just be idle and then the mind is the devil's workshop.*"

Some highlighted the influence of personal relationships and emphasized the importance of social support and emotional well-being in promoting an active lifestyle and discouraging sedentariness, stating, "*Even an unhappy marriage can also cause it.*" *(FGD6 Speaker F, Female 30 years)* Additionally, a participant raised the significance of occupational factors, and the role of workplace dynamics and job demands in shaping physical activity patterns and contributing to sedentariness, stating, "*Your position in the office can also cause sedentariness. So, it's not only poverty, it happens to people that are rich.*" *(FGD6 Speaker A, Female 26 years)*

Theme 5:

Influence of the Built Environment on Physical Activity, Sedentariness and Exercise Adoption Among Healthcare Professionals

Participants in the focus group discussions identified several significant barriers to physical activity in Lagos, focusing primarily on the built environment, safety concerns, and the availability of suitable spaces for exercise. These barriers significantly impact the ability and willingness of individuals to engage in regular physical activity. The inadequacy of pedestrian infrastructure was a major point of discussion. Participants highlighted issues such as poorly constructed sidewalks and pathways, which pose hazards to pedestrians and exercisers. FGD1 Speaker D, Female 39 years emphasized, *"Even if you have sidewalks, cars are passing on the sidewalks. So, you don't even want to walk or exercise on the sidewalk."* Similarly, FGD1 Speaker C, Male 31 years noted, *"The way the built environment is in Lagos does not give room for plenty of time walking because you can easily be knocked down by any of these vehicles."* Poor urban planning policies that fail to consider the accessibility of recreational facilities and safe public transportation options further exacerbate these challenges, they noted.

Safety was further highlighted under the built environment across all discussions. Participants further expressed concerns about the risks associated with outdoor exercise, particularly in public spaces with inadequate lighting or high crime rates. Ensuring the safety and security of individuals who engage in outdoor physical activities, especially during early morning or evening hours, was deemed crucial. FGD2 Speaker C, Female 52 years highlighted the impact of safety concerns on physical activity levels, *"In terms of the environment, definitely, it has a great impact... It is difficult for me to say I want to wake up and take a walk... Security is a major issue in my area."* Similarly, FGD2 Speaker I, Female 42 years added, *"Going out to jog around or do some form of exercise outside, it's not safe for me in my environment."* Participants shared personal anecdotes of security incidents that deterred individuals from engaging in outdoor activities. FGD3 Speaker A, Female 53 years recounted, *"A man I know was robbed and killed. Security early in the morning is a problem. The fear of even coming out when it is not broad daylight will keep you at home and prevent you from doing any form of exercise."*

The scarcity of public parks and open spaces conducive to physical activity was another significant barrier with existing areas often occupied by market vendors or deemed unsafe. FGD3 Speaker B, Female 35 years mentioned, *"Even the few parks we have are occupied by 'agberos'. The little walkways are covered by sellers of goods. There is no structure on ground that is enforced for people to even embark on exercise."* Participants

discussed how their living environment can pose challenges to engaging in physical activity, particularly if there are limited opportunities or suitable spaces available. FGD4 Speaker B, Female 38 years stated, *"...if one is not in a good environment and you don't have where you can go out for physical activity. It could be a hindrance..."* Participants expressed a strong desire for better-designed urban environments that support physical activity and improve safety, which would significantly enhance their ability to engage in regular exercise.

The discussion also revealed insights into how environmental and social factors influence physical activity and sedentary behaviors. Air and noise pollution from generators and crowded environments were identified as deterrents to outdoor activities. FGD5 Speaker D, Female 30 years noted, *"If you are going out for exercise...I'm allergic to all that stuff. Once I go through that area, I will be disturbed. I would want to go back home so it affects even sedentary behaviour as well."* Climate-related factors such as flooding and harsh sunlight were also identified as barriers. Insecurity in the environment, coupled with social stigma and peer pressure, further discouraged individuals from engaging in physical activity.

Theme: 6

Influence of Culture on Physical Activity, Sedentariness and Exercise

The discussion delved into the influence of cultural norms on individuals' attitudes toward exercise. Participants noted societal perceptions that prioritize sedentary lifestyles over physical activity. FGD1 Speaker E, Male 40 years highlighted the stigma associated with "trekking" in Nigerian culture, reflecting broader cultural ideals regarding wealth and status, where car ownership symbolizes affluence. He stated, *"The Nigerian way is trekking. The mentality is that poor people walk, the rich drive."* This reluctance to engage in active modes of transportation discourages physical activity. Participants also noted that Nigerian culture often associates being overweight with prosperity and well-being, especially for women, and sometimes for men, where a larger body size is equated with financial success. Such cultural norms prioritize a larger appearance over fitness, discouraging individuals from engaging in physical activity. Additionally, FGD1 Speaker D, Male 39 years noted cultural practices, such as the "fattening room" in Calabar culture, that promote relaxation and delegating physical tasks rather than actively pursuing fitness before marriage. This cultural preference for sedentary behaviors poses a significant barrier to exercise adoption among healthcare professionals.

FGD2 Speakers B to G offered their perspectives on how cultural norms, societal expectations, and generational shifts influence individuals' attitudes and behaviors towards exercise. FGD2 Speaker C, Female 52 years highlighted the dynamic nature of culture, noting a shift towards greater awareness of the importance of physical exercise for health in Nigeria. However, cultural perceptions of body image and modesty were identified as potential barriers, with concerns raised about societal judgment based on attire and appearance while exercising. *"I see some excesses because I see people who are going around exercising... Maybe the way they are dressed is so provocative... I also see people looking at those who are trying to exercise and wondering why they are punishing themselves." (FGD2 Speaker C, Female 52 years) "Culture is actually not encouraging exercise... An average young lady that gets married, they expect you to become fat and flourishing." (FGD2 Speaker I, Female 42 years)*

Participants noted a gradual cultural shift towards embracing exercise as a lifestyle choice, particularly among younger generations. FGD2 Speaker B, Female 45 years highlighted the increasing integration of exercise into cultural practices, such as church programs promoting aerobics sessions. *"People have become... have come to accept that exercise is good... We talk about exercise, exercise, exercise... People are beginning to imbibe exercise as a way of life." (FGD2 Speaker B, Female 45 years)* However, concerns about societal acceptance and cultural norms regarding body image persisted.

The discussion also touched upon the influence of social class on perceptions of exercise, with disparities observed in access and acceptance based on class. FGD2 Speaker F, Male 42 years underscored the perception of exercise as a foreign concept among certain social strata, highlighting the need for broader cultural acceptance and accessibility. *"Exercise is skewed to a group of people or a social class. It's observed more by the middle and upper class. The poor are still looking for how to feed." (FGD2 Speaker F, Male 42 years)*

Participants pointed out the role of age in shaping attitudes towards exercise, with younger individuals exhibiting greater enthusiasm and awareness compared to older generations. FGD2 Speaker D, M47 highlighted the transition in exercise priorities across different life stages, emphasizing the influence of health concerns and external motivators on sustaining physical activity as we age. *"Demography in terms of age does play a significant role... Exercise is the least thing that will come to mind for older people except there's an external force like a health challenge." (FGD2 Speaker D, Male 47 years)*

They suggested that cultural norms, such as societal expectations around childcare and household duties, may deter individuals, particularly women, from prioritizing exercise. *"We that we have small, small children... You can't leave your baby and go." (FGD3 Speaker F, Female 55 years)* Cultural perceptions can discourage individuals, particularly women, from engaging in exercise, associating weight loss with negative connotations such as marital issues or illness. *"So, if you are starting to exercise... they'll say maybe your husband is not feeding you well. Even your husband will start complaining if you lose too much weight."(FGD3 Speaker F, Female 55 years)*

They shared cultural practices surrounding postpartum care, where weight gain is encouraged through special diets and limited physical activity, perpetuating sedentary behaviors among women. *"If you deliver, they will make pap in a bucket... She will take it... You will see them very fat." (FGD3 Speaker E, Female 38 years)* Some cultures have traditions like "fattening rooms," where individuals are encouraged to gain weight before marriage, reinforcing societal expectations around body size and shape. *"Some cultures have fattening rooms. It's part of our culture." (FGD3 Speaker A, Female 53 years)*

They discussed how cultural norms often equate weight gain with good health and well-being, especially within certain Nigerian communities. Women may feel pressured to gain weight to conform to societal expectations of beauty and marital happiness. *"...They do things that will make them gain weight. If they want travel back to their village, they'll start gaining weight to show their husband is taking care of them." (FGD4 Speaker D, Female 35 years)* Women who do not meet these expectations may experience stigma and questioning from peers and family

members. *"...You're coming back for August meeting and you're looking skinny... they assume you have HIV." (FGD4 Speaker B, Female 38 years) "...What's wrong with this one? Is your husband not taking care of you? Why are you so slim?" (FGD4 Speaker D, Female 35 years)*

Participants also discussed how religious beliefs and practices can also influence individuals' engagement in physical activity, particularly among women who wear the hijab. The expectation of modesty may deter them from participating in outdoor exercise activities. *"...Some of these people wearing hijab, don't expect them to exercise, except if it's indoor." (FGD4 Speaker C, Female 31 years) "When it's a Muslim or Christian, you know, if you want to do exercise, there are some kind of clothes you wear, and people will start complaining." (FGD5 Speaker E, Female 48 years) "That kind of religion won't allow you to do exercise." (FGD5 Speaker D, Female 30 years)*

Societal norms and gossip, they noted, further contribute to the pressure on individuals, especially women, to conform to cultural ideals of beauty and health. Negative comments or assumptions about weight loss may prompt individuals to take drastic measures to gain weight. *"...Maybe gossiping around influence most women to do things that will make them gain weight." (FGD4 Speaker E, Female 39 years) "Some cultures believe if a woman is not chubby and you are not fat, you are not healthy...they will start raising a lot of questions." (FGD5 Speaker C, Female 22 years)*

"When somebody sort of loses weight, they say, ah, her husband is not taking care of her…what is happening? They've fallen on bad times. The person has HIV." (FGD5 Speaker A, Female 53 years)

According to the participants, cultural expectations regarding gender roles within marriage also impact physical activity levels. In some cultures, women are expected to prioritize household duties over exercise, leading to a sedentary lifestyle, while men are often encouraged to engage in physical labor. *"They are mostly housewives and do as little physical activity as possible except taking kids to school cooking and that's all." (FGD5 Speaker E, Female 48 years)* Cultural norms that prioritize sedentary lifestyles or discourage individuals, particularly women, from engaging in physical activities, they noted, also contribute to low exercise adoption. *"Some cultures encourages sedentariness. Their wives, they don't work." (FGD5 Speaker C, Female 22 years)* FGD6 Speaker C, Female 27 years highlighted the cultural perception of body size, stating, *"African culture promotes being overweight to a large extent. We like it when people are heavier. They will say you are looking "orobo". We appreciate, especially for women, when they are looking "full"."*

Theme: 7

Influence of Healthcare Organizations on Physical Activity, Sedentariness and Exercise Adoption Among Healthcare Professionals

Participants discussed the significant impact of organizational environments on physical activity levels among healthcare professionals. FGD1 Speaker B, Female 35 years highlighted disparities between workplaces, noting that while multinational organizations implemented policies promoting exercise breaks and encouraged stair use through intentional measures like disabling lifts, others lacked such initiatives entirely. Some participants pointed out the

observation that while organizations may promote physical activity promoting activities, individuals still must make personal agency to take advantage of these activities. This suggests that while organizational structures can influence behavior, personal agency remains paramount in determining exercise habits. This was articulated by FGD1 Speaker E, Male 40 years: *"In as much as the work environment can affect physical activity and has a role to play, individual decision is still a big factor. My specialty is sedentary because I sit down seeing patients all day, but I make the decision to stand up every hour".*

Theme 8:

Adaptation Strategies to Identified Barriers

Despite these barriers, participants discussed strategies they employ to overcome them. Some participants resort to exercising within the confines of their homes, utilizing videos or simple exercises that require minimal space. *"The only way out is using my sitting room... Put the video on, then I'll do the exercise." (FGD4 Speaker B, Female 38 years)* Others explored alternative locations for exercise, such as nearby gyms or parks, even if they require travel outside their immediate vicinity. *"I can always go in the evening with my son... Then we'll come back together. Because he's on the fat side, so he's motivated to exercise." (FGD4 Speaker B, Female 38 years)*

Some participants emphasized the importance of personal agency and intentionality in maintaining physical activity. FGD2 Speaker E, F50 shared their personal motivation stemming from a family history of hypertension and stroke in a parent, highlighting how setting fitness goals and monitoring blood pressure drove their commitment to regular exercise. He said this perspective underscores the role of internal motivation in overcoming environmental barriers. *"A lot also has to do with our intentionality, our desire to actually make physical activity, exercise as part of our desired goal... So throughout my medical school days, I was always jogging around despite my environment." (FGD2 Speaker E, Female 50 years) "The environment, yes, may not be so conducive... but I shouldn't be using that as an excuse... I just need more motivation." (FGD2 Speaker B, Female 45 years)*

Theme 9:

Recommendations for Organizational Policies to Increase Physical Activity, Promote Exercise and Reduce Sedentariness

Participants discussed various strategies that healthcare organizations could implement to promote physical activity among staff. FGD1 Speaker F, Female 38 years suggested implementing policies similar to those in multinational companies, encouraging regular breaks for physical activity. FGD1 Speaker E, Male 40 years emphasized the importance of health education and awareness campaigns within healthcare settings to promote the benefits of exercise. Additionally, FGD1 Speaker C, Male 31 years advocated for the creation of conducive environments for physical activity, including on-site gyms and integrating exercise into the organizational culture. They emphasized the need for proactive measures such as designated exercise hours, provision of recreational facilities, and incentivized participation. One participant noted, *"If the organization's leadership can make it important, many people will be encouraged to be involved." (FGD5 Speaker D, Female 30 years)*

They proposed solutions such as free gym memberships for staff, incentivized health checks tied to exercise attendance, and tailored messaging to enhance motivation. FGD1 Speaker D, Female 39 years suggested placing signage and informational materials at strategic parts of the healthcare organization to inspire exercise and discourage sedentariness. FGD1 Speaker A, Female 41 years suggested the use of incentives, stating, *"Incentives always work. Something as small as bread can motivate people. If we want to incentivize exercise, it could be as simple as offering discounts on healthy snacks or providing free gym memberships."*

Despite existing challenges, participants expressed optimism about the future of physical activity among healthcare professionals in Lagos State. They emphasized the need for increased awareness, education, and advocacy to drive sustainable behavior change. FGD1 Speaker B, Female 35 years remarked, *"The future is bright, but we need to do more."* FGD1 Speaker D, Female 39 years added, *"With repeated awareness and individual action, we can spark the change needed to promote a culture of physical activity within our healthcare facilities."*

FGD2 Speaker C, Female 52 years advocated for the establishment of onsite fitness centers equipped to meet the needs of employees. Emphasizing accessibility and affordability, she proposed subsidies for staff members or partnerships with nearby fitness centers to incentivize engagement in physical activity, stating, *"I would like to ensure that there's a proper fitness center... well trained staff, and then some level of subsidy... because prevention is better than cure."* FGD3 Speaker D, Female 32 years stated, *"If they said if you come today, they will give you something, you know, people will be rushing to come..."* Still on incentives, proposals were made for linking health-related benefits to individuals' BMI, incentivizing healthy weight management and encouraging participation in physical activity. FGD3 Speaker B, Female 35 years suggested, *"If there is an enforcement of a particular BMI that one should be able to attain according to your age that will affect your promotion, it might help some people."*

FGD2 Speaker G, Female 46 years stressed the significance of leadership involvement in promoting physical activity through role modeling and engagement initiatives. Drawing inspiration from university practices, they suggested regular walks led by top management as opportunities for social networking and role modeling, fostering a culture of physical wellness within the organization. *"Make that time a safe space… to encourage that engagement." (FGD2 Speaker G, Female 46 years)*

FGD2 Speaker F, Female 46 years advocated for the enforcement of physical activity policies through the implementation of rosters and penalties for non-compliance, emphasizing the necessity of accountability measures to drive participation, with clear expectations and consequences. *"People don't like to do this thing called exercise... You put up a roster and enforce it."(FGD2 Speaker F, Male 42 years)* FGD2 Speaker D, Male 47 years disagreed, proposing the promotion of persuasive benefits and incentives rather than coercion to encourage exercise participation. He emphasized the importance of framing physical activity as a compelling choice rather than a mandatory requirement, suggesting competitions and awards as motivational tools to drive engagement. *"You have to put a compelling reason to exercise so they can see the advantages." (FGD2 Speaker D, Male 47 years)*

Participants also highlighted the role of support groups and attitudinal changes in fostering exercise adoption. FGD2 Speaker B, Female 45 years emphasized the importance of serving as role models, while FGD2 Speaker C, Female

52 years stressed the significance of collective efforts in driving attitudinal shifts towards physical activity. *"Working out together... can help that attitudinal change."* (FGD2 Speaker D, Male 47 years) Stressing the importance of accessible gym facilities within healthcare facilities to enable healthcare workers to engage in physical activity conveniently during breaks or at designated times, FGD3 Speaker C, Female 25 years stated, *"They can create a gym in our healthcare facilities if they want us to exercise so that we can go there when we are not busy, then come back to work."*

Addressing the need for adequate staffing levels, participants emphasized the importance of having enough personnel to cover duties to allow individuals to take breaks for physical activity without compromising patient care. FGD3 Speaker A, Female 53 years noted, *"There have to be more health workers on the ground... If we have enough staff, you can say, okay, you are around, let me go for 30 minutes. You can cover up and then maybe when it's your turn, I can always cover up for you while you go."*

Participants stressed the importance of creating widespread awareness about the benefits of physical activity, advocating for community-wide initiatives and educational campaigns to instill an exercise mentality. FGD3 Speaker B, Female 35 years stated, *"Everybody should have the exercise mentality... From the community level to the states to the top, everybody must have that exercise mentality."* Recommendations were made for organizing community health missions and outreach programs to provide access to BMI assessments and health checks, raising awareness about the importance of physical activity and its impact on health. FGD3 Speaker A, Female 53 years mentioned, *"There should be community health missions, community health outreaches, whereby people will have their BMI checked... At least it will give them that sense of awareness that they should start exercising."*

FGD6 Speaker F, Female 30 years proposed the creation of designated exercise times and spaces within healthcare facilities, stating, *"They can create a time for us... maybe they are going to group us in batches and have professionals to train us."* FGD6 Speaker A, Female 26 years emphasized the role of awareness and education in promoting physical activity. She underscored the importance of disseminating information about the benefits of physical activity to motivate healthcare professionals to adopt active lifestyles, stating, *"They should create more awareness and display the benefits of physical activity and exercise. If they can do that, it will really help health workers."*

Furthermore, FGD6 Speaker D, Female 35 years highlighted the importance of leadership support and encouragement. She noted the influential role of leadership in setting a positive example and motivating staff to prioritize physical activity, stating, *"My boss, she's very active, very agile. Seeing her doing many things... encourages us."* Additionally, FGD6 Speaker A, Female 26 years emphasized the importance of supportive supervision and accountability, stating, *"If you supervise people properly, whatever you ask people to do, they will do it. You supervise in a friendly manner. That is motivation and encouragement."*

Summary of the Themes

Personal Experiences

Participants shared their personal experiences with physical activity, highlighting struggles with maintaining regular exercise routines due to time constraints, environmental and motivational challenges. Success stories included teaming up with peers or hiring fitness instructors to facilitate adherence to exercise programs.

Barriers to Physical Activity

Barriers included environmental challenges like poor pedestrian infrastructure, safety concerns, and lack of recreational spaces. Cultural barriers included societal perceptions equating car ownership with wealth and associating being overweight with prosperity and discouraging physical activity. Difficulty maintaining motivation, especially without supportive environments was repeatedly highlighted.

Facilitators of Physical Activity

Implementing policies encouraging regular breaks for physical activity, establishing onsite gyms, and integrating exercise into the organizational culture was proposed. Leadership involvement through role modeling and engagement initiatives, team-based exercises, and hiring fitness instructors were seen as key facilitators. Provision of free gym memberships, discounts on healthy snacks, organizing competitions, awards and other incentives were proposed.

Factors Contributing to Sedentary Behaviour

Cultural norms associating being overweight with prosperity, and equating car ownership with wealth were major contributors to sedentary behaviour, according to the participants. Cultural practices such as the "fattening room" in Calabar culture further promote sedentary behavior. Lack of designated exercise hours, inadequate staffing levels preventing breaks, and limited access to exercise facilities at healthcare organizations enabled sedentariness in the view of the participants. Traffic challenges in Lagos State was also seen as a major contributor to sedentariness due to the length of time that commuters spent sitting in vehicles.

Influence of the Built Environment

Participants emphasized that the built environment, with poor pedestrian infrastructure and lack of recreational spaces, significantly impacted their ability to engage in physical activity. Unsafe sidewalks and insufficient lighting in public areas were major deterrents. The poor security situation in the city also made it unsafe for participants to venture outside their homes at certain times for exercise.

Influence of Healthcare Organizations

Healthcare organizations had a significant influence on physical activity levels among staff, the participants noted. Organizational policies that support physical activity, such as providing on-site gyms and encouraging regular exercise breaks, could facilitate exercise adoption, in their view. Leadership involvement and the creation of a supportive environment were also considered crucial.

Adaptations to Barriers

Despite all the identified barriers, the exercise adopters were able to share various ways in which they adapted to these barriers. Using videos or simple exercises that require minimal space proved successful for some. For others, traveling to nearby gyms or parks for exercise were options that worked. Forming support groups and leveraging peer motivation to maintain exercise routines proved beneficial for many.

Recommendations for Organizational Policies

Their recommendations included ensuring exercise facilities are accessible and affordable for all staff. Provision of subsidies for fitness center memberships and partnerships with nearby gyms were recommended. Scheduling structured exercise programs tailored to individual needs, with professional trainers was suggested by those that had health challenges. Another suggestion was the creation of designated exercise times and spaces within healthcare facilities. Increasing awareness about the benefits of physical activity through educational campaigns within healthcare facilities was seen as vital. Offering monetary rewards and incentives for engaging in physical activity, attending gym sessions or achieving health-related goals was proposed. In addition to the above, implementation of supportive supervision and accountability to encourage physical activity within organizations and encouraging leaders to set a positive example by participating in physical activities and promoting a culture of wellness were proposed.

S 6 TABLE: SOCIODEMOGRAPHIC CHARACTERISTICS OF IN-DEPTH INTERVIEW (IDI) AND KEY INFORMANT INTERVIEW (KII) RESPONDENTS

Interviews	Respondents	Age	Sex	Years in Position	Relationship to HCPs/Managerial Position
**In-Depth Interviews – Interpersonal**					
	Respondent 1	56	Male	10*	Spouse
	Respondent 2	45	Male	12*	Spouse
	Respondent 3	47	Male	14*	Spouse
	Respondent 4	28	Male	28*	Sibling
**Key Informant Interviews – Organizations**					
	Manager 1	45	Male	5	Medical Director – General Hospital
	Manager 2	59	Female	12	Medical Director – General Hospital

	Manager 3	51	Male	5	Chairman Medical Advisory Council (CMAC) – Teaching Hospital
**Key Informant Interviews – Policy Makers**					
	Policymaker 1	45	Female	4	Chief Town Planning Officer
	Policymaker 2	46	Female	2	District Director, Medical Services and Disease Control

***Years of relationship**

In-Depth Interview Report – Interpersonal Level

The in-depth interview report was structured in alignment with the research objectives to provide a comprehensive analysis of the findings.

Theme 1: Perceived Level of Physical Inactivity Among HCPs

The prevalence of physical inactivity among healthcare professionals was highlighted across all interviews. While the professionals understand the benefits of regular exercise, most do not engage consistently. Respondent 2, for instance, described their healthcare professional spouse as having a sedentary lifestyle despite a supportive environment and occasional encouragement to exercise. Respondent 4 shared that their healthcare professional relative was motivated to begin exercising after purchasing a treadmill, yet they have not sustained regular activity levels. In many cases, physical inactivity appeared to be linked to work-related fatigue, as noted by one respondent:

> *"She knows the benefits of exercise, but after a long day at work, she feels too drained to do anything." (Respondent 1, Male, 56 years)*

Sedentariness is a significant concern, with participants reporting long periods of sedentariness during both work and leisure. Healthcare professionals often spend their free time engaged in passive activities such as phone usage,

watching television, or resting after shifts. Respondent 3 observed that their healthcare professional relative has a sedentary lifestyle at home, stating:

> *"After work, she prefers to rest or use her phone instead of exercising." (Respondent 3, Male, 47 years)*

Respondent 2 noted that even at work, healthcare professionals may experience prolonged periods of standing or sitting, further contributing to their overall sedentary behaviour. Respondents believed that the physical demands of healthcare work, coupled with limited energy during downtime, exacerbate sedentariness.

Theme 2: Determinants of Physical Inactivity and Sedentariness

Work schedules and family responsibilities were identified as key deterrents to physical activity. They bemoaned the fact that their healthcare professional relatives often work long hours under stressful conditions, leaving little time or energy for exercise. Respondent 3 explained that balancing family duties with work responsibilities creates significant challenges, stating:

> *"She barely has time for herself, let alone physical activity." (Respondent 3, Male, 47 years)*

Fatigue and low energy levels were recurring themes across interviews, with Respondent 2 noting that while their spouse has time for exercise,

> *"Mental exhaustion from work makes it hard for her to feel motivated to exercise." (Respondent 2, Male, 45 years)*

Lack of motivation among their HCP family members was another issue frequently highlighted by respondents. Respondent 4 mentioned that their relative, despite wanting to be more active, often struggles with initiating or maintaining regular exercise routines. The influence of cultural and social on physical activity behaviours was also alluded to. While none of the respondents identified cultural barriers to exercise, Respondent 2 remarked that physical activity is not traditionally emphasised in their community. However, Respondent 4 pointed out that their family's cultural practices during festivals, which involve active participation in events, positively influence physical activity habits and promote active lifestyles.

Social factors also play a role. The absence of peers or colleagues who prioritise physical activity was noted as a potential deterrent. One respondent observed:

> *"The healthcare professional does not have friends who regularly exercise, which might contribute to her physical inactivity." (Respondent 2, Male, 45 years)*

The consensus was that the lack of socially active peer groups may limit opportunities for healthcare professionals to engage in group fitness activities or receive encouragement from their immediate social network.

Theme 3: Facilitators of Exercise Adoption

Despite the challenges, several facilitators of exercise adoption were identified. Family support emerged as a critical enabler. Respondent 4 explained that their family regularly invites the healthcare professional to participate in activities such as jogging, gym sessions, and walking, which have contributed to improving her physical strength. Spousal support was particularly effective, with Respondent 1 noting that shared activities, such as gym visits or evening walks, have helped their spouse remain somewhat active.

Cultural norms, while not universally influential, were also noted to serve as facilitators. Respondent 4 shared that their family's traditions of incorporating physical activities into festivals encourage a mindset where exercise is seen as enjoyable and meaningful. Flexible work hours, where available, were another facilitator mentioned by respondent. Respondent 2 suggested that adjusted schedules could create more time for physical activities, especially for professionals with demanding workloads.

Community programmes designed to promote physical activity were identified as valuable resources. Respondent 1 proposed hospital-organised initiatives to educate healthcare professionals on the importance of fitness while also reducing workload pressures. They believed that these programmes, combined with family encouragement and social support, could provide a comprehensive framework for fostering exercise adoption.

Theme 4: Barriers to Exercise Adoption

Barriers to exercise adoption were consistently highlighted across all interviews. Time constraints due to long work hours and family responsibilities were the most significant obstacles. Respondent 3 emphasised that healthcare professionals often prioritise work and caregiving roles over self-care, stating:

> *"It's not just about having time; they also need energy and the right mindset to engage in physical activity." (Respondent 3, Male, 47 years)*

Fatigue and low energy levels further exacerbate this issue, with Respondent 2 describing how physical and emotional exhaustion often deter their spouse from exercising, even when time is available.

Motivational challenges were also identified as impeding regular exercise. Respondent 4 noted:

> *"She wants to be more active but struggles with the discipline to stick to a routine." (Respondent 4, Male, 28 years)*

Limited access to gym facilities or suitable environments for outdoor exercise was occasionally mentioned as a barrier. Respondent 1 added that the availability of fitness centres could influence participation, particularly for those who require structured settings for exercise.

Theme 5: Recommendations for Supporting Exercise Adoption

The findings highlight the importance of addressing both structural and interpersonal factors to support healthcare professionals in adopting regular physical activity. Families can play a significant role by creating an environment conducive to exercise. Respondent 4 suggested organising group activities, setting shared fitness goals, and offering assistance with household responsibilities to free up time for physical activity. Hospitals and workplaces could also implement flexible schedules and wellness programmes that prioritise fitness. Respondent 1 proposed that hospitals organise initiatives to reduce workload pressures, stating:

> *"Such programmes would help healthcare professionals find time for physical fitness." (Respondent 1, Male, 56 years)*

Promoting peer influence and social engagement was another key recommendation. Respondent 2 highlighted the potential of reconnecting healthcare professionals with physically active colleagues or friends to foster a sense of camaraderie and motivation. Community-based fitness programmes tailored to healthcare professionals' schedules could further encourage participation.

Finally, they proposed that cultural and social frameworks should be leveraged to normalise and encourage physical activity. Respondent 4's experience with culturally embedded physical activities illustrates the potential of integrating exercise into social events and traditions.

Summary

The interviews revealed a complex interplay of factors influencing physical inactivity, sedentariness, and exercise adoption among healthcare professionals in Lagos State from the perspectives of their closest family members. While barriers such as demanding work schedules, fatigue, and motivational challenges persist, facilitators such as family support, spousal involvement, and cultural influences offer pathways to improvement. By addressing these barriers and leveraging existing facilitators, healthcare professionals can be better supported to adopt and maintain regular physical activity, contributing to improved physical and mental wellbeing.

Key Informant Interview Report – Organisational Level

The findings are structured based on the research objectives:

Theme 1: Perceived Level of Physical Inactivity Among HCPs

All participants emphasised the widespread prevalence of physical inactivity among healthcare professionals in Lagos State. The primary contributors were high workloads and time constraints.

One manager stated:

> *"Our staff are so overburdened with their patient loads that exercise becomes an afterthought. In some clinics, doctors see over 30 patients daily. Where would they find the time for physical activity?" Manager 1, Male, 45 years*

Another manager highlighted a similar trend:

> *"Since the facility's renovation increased our patient capacity, the workload has skyrocketed, leaving little time for staff to prioritise exercise." Manager 2, Female, 59 years*

Another manager pointed to the post-COVID-19 workload as a significant driver of inactivity:

“The pandemic created a situation where staff were stretched to their limits. Even now, we’re still understaffed, which means fewer people are doing more work, and physical activity is one of the first things they abandon.” Manager 3, Male, 51 years

The same manager also pointed to the persistent “japa” syndrome as responsible for the dearth of healthcare professionals, compounded by low recruitment numbers due to inadequate human resource budgets. In all, responses indicated that while some healthcare professionals might be aware of the benefits of exercise, the high demands of their jobs significantly limit their ability to engage in physical activity.

Sedentariness was particularly noted among non-surgical and administrative healthcare professionals. The long hours of sitting and lack of structured interventions to combat this behavior have exacerbated the problem according to the respondents. One manager observed:

“In outpatient and administrative roles, healthcare professionals spend most of their shifts seated. This sedentary lifestyle is as much of a problem as the lack of exercise itself.” Manager 3, Male, 51 years

One manager added:

“Even roles that involve some movement are not active enough to combat the negative effects of sedentariness. Without deliberate interventions, these habits persist.” Manager 2, Female, 59 years

Theme 2: Determinants of Physical Inactivity and Sedentariness

The most significant determinant of inactivity across all interviews was the demanding workload and inadequate staffing levels. One manager noted:

“The sheer number of patients our staff are expected to handle daily leaves them drained. It’s not just about time—it’s the exhaustion they feel after their shifts.” Manager 1, Male, 45 years

“We’re struggling with understaffing, and this means healthcare professionals are multitasking and working longer hours. It’s hard to think about exercise when you barely have time for a break.” Manager 2, Female, 59 years

The lack of designated spaces for physical activity was another factor identified.

"The renovations improved patient care spaces but didn't consider staff wellness. We don't have a dedicated gym or even a proper area where staff can unwind or exercise." Manager 2, Female, 59 years

Another manager echoed this sentiment:

"While we've set aside some space for activities, it's not equipped to meet the needs of a diverse group of professionals." Manager 1, Male, 45 years

The managers also noted that physical activity was not deeply embedded in the organisational culture of their organisations.

"There's no policy or systemic encouragement for exercise. We have to change the mindset and create a culture where physical activity is seen as essential, not optional." Manager 3, Male, 51 years

Theme 3: Facilitators of Exercise Adoption

The participants proposed several strategies to encourage exercise adoption among healthcare professionals. Increasing awareness of the benefits of exercise and the risks of inactivity was a recurring theme.

"A lot of healthcare workers know exercise is good for them, but consistent reminders through wellness talks or campaigns can help keep it on their radar." Manager 3, Male, 51 years

Appointing leaders within departments to promote exercise as exercise champions was seen as a likely facilitator.

"If every department had a champion for physical activity, it would create a ripple effect. People follow what they see their leaders doing." Manager 1, Male, 45 years

Using fitness trackers and mobile apps to encourage physical activity and track progress was suggested.

"These tools can make exercise engaging. Imagine friendly competitions among departments based on step counts—this can motivate even the busiest professionals." Manager 3, Male, 51 years

Collaboration with external organisations was another idea.

"Registering staff at clubs for activities like swimming or tennis, with discounts for group registrations, could make exercise more accessible." Manager 2, Female, 59 years

Theme 4: Barriers to Exercise Adoption

The most frequently mentioned barrier was the lack of time and energy due to demanding work schedules.

> *"The long shifts and high patient load leave staff physically and mentally drained. Even when they have time, they don't have the energy to exercise." Manager 1, Male, 41 years*

> *"It's not that they don't want to exercise; it's that they are completely exhausted by the time their shifts end." Manager 2, Female, 59 years*

The absence of policies and structured programs to promote physical activity was another major barrier.

> *"Without official backing, these initiatives feel like afterthoughts. We need clear policies to prioritise staff wellness." Manager 3, Male, 51 years*

Another manager added:

> *"Even if we want to promote exercise, the lack of resources—like a proper gym—limits what we can achieve." Manager 2, Female, 59 years*

Theme 5: Recommendations for Addressing Physical Inactivity

Incorporating brief physical activity breaks during work shifts was a recommended solution to encourage healthcare professionals to engage in exercise without disrupting their demanding schedules. These short, structured activities could provide a practical solution to combat sedentariness and improve overall well-being in a high-pressure work environment. One manager highlighted this strategy, stating:

> *"Even a five-minute stretch or walk during breaks can make a difference." Manager 2, Female. 59 years*

Leveraging technology and gamification was another recommendation. By introducing fitness apps and trackers, healthcare facilities can foster a culture of healthy competition and engagement among staff. One manager emphasised the potential of this approach, noting:

> *"Staff are more likely to engage when there's an element of fun or competition." Manager 3, Male, 51 years*

Such tools can track activity levels, encourage goal setting, and create camaraderie among colleagues through friendly competitions.

Recognition and incentive programs were also suggested as tools that can play a crucial role in motivating staff to adopt an active lifestyle. One manager suggested rewarding participants with tokens of appreciation, saying:

> *"Incentives like recognition plaques or even small gifts can motivate people to be more active." Manager 1, Male, 45 years*

These programs could reinforce positive behaviour and help integrate physical activity into the organisational culture. In addition, optimising existing resources was seen as a practical approach to overcoming space and equipment constraints. One manager pointed out that even with limited facilities, creativity can drive change:

> *"We don't need fancy gyms; we just need to creatively use what we have." Manager 2, Female, 59 years*

They suggested that facilities like physiotherapy departments and underutilised spaces can be repurposed for structured exercise programs.

Finally, advocating for policy support was identified as essential to ensuring sustainability and institutionalisation of wellness programs. One manager highlighted the importance of this step, stating:

> *"Policy support is essential to making these changes sustainable." Manager 3, Male, 51 years*

The respondents agreed that healthcare facilities can establish formal wellness initiatives, creating environments that prioritise the health and well-being of their staff.

Summary

This report highlights the complex interplay of workload, organisational culture, and resource limitations in shaping physical inactivity and sedentariness among healthcare professionals in Lagos State. While significant barriers exist, there are also practical facilitators, such as awareness campaigns, leadership champions, and technological tools, that can drive change. By addressing these issues through targeted interventions and robust policy support, healthcare facilities can foster a culture of physical activity, improving both the health and job satisfaction of their staff.

Key Informant Interview Report – Policy Level

This report synthesizes insights from a key informant interview with two respondents: the Chief Town Planning Officer of Lagos State and the Director of Medical Services for District 6 of the Lagos State Ministry of Health. The report is structured according to the study's research objectives to explore the determinants of physical inactivity, sedentariness, and exercise adoption among healthcare professionals.

Objective 1: Perceived Level of Physical Inactivity Among HCPs

Physical inactivity among healthcare professionals in Lagos State is prevalent, as indicated by both respondents. Policymaker 1 noted that while some healthcare professionals are physically active, many struggle to prioritise exercise due to demanding work schedules and the absence of recreational facilities within healthcare establishments. Policymaker 2 corroborated this, highlighting that existing policies mandate monthly physical activity for civil servants, including healthcare professionals. However, gaps in implementation and enforcement lead to inconsistent participation.

> *"Although healthcare professionals understand the importance of physical activity, they often neglect it due to the pressures of their work and limited opportunities to exercise regularly." (Policymaker 2, Female, 46 years)*

This pattern highlights a disconnect between awareness and practice, emphasising the need for practical interventions.

Sedentariness was also a major concern, driven by both professional and urban constraints. Policymaker 1 explained that healthcare professionals in Lagos are often subjected to sedentary behaviours due to long work hours and the design of their workspaces, which do not encourage movement. This is exacerbated by the physical environment in Lagos, where traffic congestion and inadequate public transport systems force healthcare workers into prolonged periods of inactivity during commutes. Policymaker 2 noted that healthcare professionals also face a lack of designated spaces for recreational activities in their workplaces.

> *"Even when professionals are willing to be active, the environment does not support it. Facilities are either absent or inaccessible." (Policymaker 2, Female, 46 years)*

They agreed that a combination of structural and professional barriers perpetuates sedentary lifestyles among healthcare professionals.

Theme 2: Determinants of Physical Inactivity and Sedentariness

Several determinants of physical inactivity and sedentariness emerged during the interviews. Workload emerged as the most significant factor, with both respondents identifying overwork and understaffing as key issues. Policymaker 1 remarked:

> *"Healthcare professionals are so consumed by their duties that physical activity often becomes a secondary priority." Policymaker 1, Female, 45 years*

Urban planning issues were also identified as determinants. Policymaker 1 highlighted that older parts of Lagos lack sufficient pedestrian-friendly areas and recreational spaces, which discourages active lifestyles. Although newer developments incorporate public spaces and parks, their accessibility remains limited, particularly for those working in high-density areas. Safety concerns, including poor lighting and encroachment on walkways, further deter outdoor exercise.

Policymaker 2 added that institutional policies within healthcare facilities rarely prioritise physical activity.

> *"While there are general mandates for civil servants, specific efforts targeting healthcare professionals remain lacking. Without tailored policies, it is difficult to address their unique challenges." (Policymaker 2, Female, 46 years)*

Theme 3: Facilitators of Exercise Adoption Among Healthcare Professionals

Despite the barriers, several facilitators of exercise adoption were discussed. Both respondents agreed that a supportive work environment and accessible recreational infrastructure could significantly enhance physical activity levels among healthcare professionals.

Policymaker 1 pointed to ongoing efforts to integrate fitness-friendly infrastructure into Lagos's urban planning.

"Our master plan includes provisions for one stadium per local government area. Although implementation is slow, these spaces could become valuable resources for healthcare professionals." (Policymaker 2, Female, 46 years)

Policymaker 1 emphasised the importance of workplace wellness programmes. She suggested incorporating structured physical activities into healthcare institutions' schedules and leveraging national health awareness days to promote exercise.

"Healthcare facilities should be champions of active lifestyles. Organising regular fitness events can encourage participation and create a culture of wellness." (Policymaker 1, Female, 45 years)

Both respondents also recognised the potential of community-based initiatives, such as exercise groups and public health campaigns, to facilitate exercise adoption. These programmes could address broader structural barriers while fostering a sense of collective motivation among healthcare professionals.

Theme 4: Barriers to Exercise Adoption Among Healthcare Professionals

Numerous barriers to exercise adoption were identified, spanning professional, structural, and policy-related domains. The most significant professional barrier is workload.

"Healthcare professionals face immense demands, leaving them physically and mentally drained by the end of the day. Exercise often becomes an afterthought." (Policymaker 2, Female, 46 years)

Urban barriers, including traffic congestion and poor infrastructure, were also highlighted. Policymaker 1 observed:

"Even if healthcare professionals are motivated to exercise, navigating Lagos's traffic and finding safe spaces to do so can be overwhelming." (Policymaker 1, Female, 45 years)

The lack of pedestrian walkways, insufficient lighting, and air pollution were additional challenges cited. Policy implementation issues further compound these barriers. Both respondents agreed that bureaucratic delays, funding constraints, and competing priorities hinder the development and enforcement of physical activity policies.

"Leadership must take a proactive approach. Without sustained advocacy, these initiatives will remain on paper." (Policymaker 2, Female, 46 years)

Theme 5: Recommendations for Addressing Physical Inactivity

Both respondents provided actionable recommendations to address physical inactivity among healthcare professionals. Policymaker 1 emphasised the need to prioritise fitness-friendly urban infrastructure, suggesting that policies should allocate resources for parks, fitness centres, and pedestrian-friendly spaces. She added that early budget proposals could help address funding constraints and ensure timely implementation.

Policymakers 2 recommended that healthcare institutions adopt written workplace policies to combat sedentary behaviours. She suggested integrating physical activity into daily routines through initiatives like fitness breaks or after-work exercise classes. She also proposed tax incentives for private fitness centres to increase accessibility for healthcare professionals.

Both respondents agreed on the importance of collaboration.

> *"Urban planners, healthcare leaders, and public health officials must work together. Only through multisectoral partnerships can we create an environment that supports active lifestyles." (Policymaker 1, Female, 45 years)*

Summary

This key informant interview report highlights the multifaceted nature of physical inactivity and sedentariness among healthcare professionals in Lagos State. While workload, urban constraints, and policy gaps remain significant challenges, there is considerable potential for improvement through targeted interventions. By enhancing urban infrastructure, implementing workplace wellness programmes, and fostering collaborative efforts, Lagos State can create an enabling environment for healthcare professionals to adopt and sustain active lifestyles. The insights provided by the respondents highlight the importance of integrating policy changes with structural improvements to address physical inactivity comprehensively.

Overall Summary of In-Depth Interviews and Key Informant Interviews

The in-depth interviews and key informant interviews with family members of healthcare professionals, healthcare facility leaders, and policymakers in Lagos State revealed a multifaceted and complex interplay of factors influencing physical inactivity, sedentariness, and exercise adoption. Workload demands, organisational culture, urban constraints, and policy gaps emerged as significant barriers to physical activity, compounded by fatigue and motivational challenges. Healthcare professionals face long hours, limited resources, and a lack of structured wellness programmes, which exacerbate sedentariness and limit opportunities for exercise.

Despite these barriers, the interviews also highlighted several facilitators that offer pathways to improvement. Family support, spousal involvement, cultural influences, and departmental champions provide social and interpersonal frameworks to encourage physical activity. Within organisations, awareness campaigns, leadership champions, and technological tools such as fitness trackers were identified as effective mechanisms for promoting exercise. Policymakers and healthcare leaders emphasised the importance of improving urban infrastructure, enhancing resource availability, and integrating workplace wellness programmes to address these challenges comprehensively. Enhancing organisational support, fostering collaborative efforts, and creating an enabling environment can were all identified as tools to drive meaningful change.

Links to FGD, IDI and KII Audio

Doctors – Exercise

https://blossom-ky6x.dovetail.com/projects/15LpgttRJYmrOTCJfbDB6J

Doctors – No Exercise

https://blossom-ky6x.dovetail.com/projects/6ghkWQD68gMs5oU9sbyhrD

Nurses - Exercise

https://blossom-ky6x.dovetail.com/data/3lzwVsaXyMD9BLM7APWkRm

Nurses – No Exercise

https://blossom-ky6x.dovetail.com/projects/5nXzqSaUHbYx0UodLHNVw3

CHEWs/CHOs - Exercise

https://blossom-ky6x.dovetail.com/data/6fNtVNl7iud4De29UPHoGd

CHEWs/CHOs – No Exercise

https://blossom-ky6x.dovetail.com/projects/6l2PuGORAyUnwqOINkVQZ3

IDIs

https://blossom-ky6x.dovetail.com/data/3GmnJYFy2ZVOi99MBdcr0I

https://blossom-ky6x.dovetail.com/f/1wJBwgMQqI3OMLYinufrg9

https://blossom-ky6x.dovetail.com/f/4vFfnCp5zdDrUyAlPNjRNI

https://blossom-ky6x.dovetail.com/data/7eU9uDloHCXSAHVJ7PbYT0

KIIs

https://blossom-ky6x.dovetail.com/f/4MdOWgQfbpd74hGOKizj1s

https://blossom-ky6x.dovetail.com/f/3mt9APRB6tOzdyuZmdV26Q

https://blossom-ky6x.dovetail.com/f/54B9kqM4NcIpDnTaJQ4f90

https://blossom-ky6x.dovetail.com/f/7yp4VTNda7TNpKWd2AytIz
