# Supplementary material for: Facilitators and barriers of physical activity, sedentariness and exercise adoption among healthcare professionals in Lagos State, Nigeria – A qualitative review
Source: PLoS One. 2026 Feb 3;21(2):e0342108. doi: 10.1371/journal.pone.0342108 (PMC12867225; doi:10.1371/journal.pone.0342108)
Supplement: Attachment — Submitted filename: Point to Point Response BM 2.pdf [file pone.0342108.s002.pdf]

Dear Editor,

We sincerely thank the Reviewers for their thorough evaluation of our manuscript and for the insightful, constructive comments provided. These comments have greatly improved the clarity, rigor, and overall quality of the paper. We have carefully considered each suggestion and have revised the manuscript accordingly. Below, we provide a point-by-point response, addressing all queries and indicating where corresponding changes have been made in the revised manuscript. All modifications are clearly marked, and we trust that the revised version satisfactorily meets the journal's expectations. We remain grateful for the time and expertise invested in strengthening our work.

Sincerely,

Authors

Point to Point Response

1. Please submit a fully or substantially completed COREQ or SRQR Checklist as this is a journal requirement for all Qualitative studies.

Response: This has been done.

2. Kindly submit copies of the two or all Ethical approval letters from all Research Ethics Committees that approved this study.

Response: This has been done.

3. Please ensure that your manuscript meets PLOS ONE's style requirements, including those for file naming. The PLOS ONE style templates can be found at

https://journals.plos.org/plosone/s/file?id=wjVg/PLOSOne_formatting_sample_main_body.pdf
and

https://journals.plos.org/plosone/s/file?id=ba62/PLOSOne_formatting_sample_title_authors_affiliations.pdexf

Response: This has been done.

4. Thank you for stating the following in your Competing Interests section: "No competing interests are declared."

Please complete your Competing Interests on the online submission form to state any Competing Interests. If you have no competing interests, please state "The authors have declared that no competing interests exist.", as detailed online in our guide for authors at http://journals.plos.org/plosone/s/submit-now
This information should be included in your cover letter; we will change the online submission form on your behalf.

Response: This has been done.

5. In the online submission form, you indicated that [All relevant data supporting the findings of this study are contained within the manuscript. Additional anonymized transcripts and qualitative datasets may be made available upon reasonable request to the corresponding author (Dr. Blossom Adaeze Maduafokwa, bmaduafokwa@yahoo.com)].
All PLOS journals now require all data underlying the findings described in their manuscript to be freely available to other researchers, either 1. In a public repository, 2. Within the manuscript itself, or 3. Uploaded as supplementary information.
This policy applies to all data except where public deposition would breach compliance with the protocol approved by your research ethics board. If your data cannot be made publicly available for ethical or legal reasons (e.g., public availability would compromise patient privacy), please explain your reasons on resubmission and your exemption request will be escalated for approval.

Response: Data supporting the findings of this study are now presented in the supplementary file. The qualitative findings are based on focus group discussions, in-depth interviews, and key informant interviews conducted with healthcare professionals, their family members, healthcare leaders and policymakers.

Due to the sensitive nature of the qualitative data, which include detailed personal experiences, workplace practices, and professional roles, full public deposition of verbatim transcripts could risk deductive disclosure and compromise participant confidentiality, in accordance with the protocol approved by the research ethics committee.

To ensure transparency while maintaining ethical compliance, we have:

- **Provided excerpts of anonymized participant quotations within the manuscript to support all themes and findings.**
- **Uploaded the interview guides, coding framework, and thematic structure as supplementary materials to allow reproducibility and methodological scrutiny.**

6. Your ethics statement should only appear in the Methods section of your manuscript. If your ethics statement is written in any section besides the Methods, please move it to the Methods section and delete it from any other section. Please ensure that your ethics statement is included in your manuscript, as the ethics statement entered into the online submission form will not be published alongside your manuscript.

Response: Thank you for this observation. The correct Ethical approval number HREC No: ADM/DSCST/HREC/APP/4115 has been indicated on page 12, line 44 only.

7. Have the authors made all data underlying the findings in their manuscript fully available? The PLOS Data policy requires authors to make all data underlying the findings described in their manuscript fully available without restriction, with rare exception (please refer to the Data Availability Statement in the manuscript PDF file). The data should be provided as part of the manuscript or its supporting information, or deposited to a public repository. For example, in addition to summary statistics, the data points behind means, medians and variance measures should be available. If there are restrictions on publicly sharing data—e.g. participant privacy or use of data from a third party—those must be specified.
Response: This has been addressed above.

8. Ethics approval inconsistencies
There are two different HREC approval numbers in the manuscript. Please correct the record and

ensure the exact name of the committee, the single valid approval number, and the date of approval are clearly stated. Confirm that procedures described (audio-recording, consent process, data use) match the approved protocol.

Response: Thank you for this observation. The correct Ethical approval number HREC No: ADM/DSCST/HREC/APP/4115 has been indicated on page 12, line 44 only.

9. Recruitment, sampling, and saturation need more detail
The manuscript states purposive sampling and that sample size was "guided by saturation," but it lacks detail on:

• How participants were recruited (gatekeepers, public postings, direct invitations).

• Numbers invited vs. enrolled, and reasons for non-participation (if known).

• What constituted your saturation criterion and when it was reached.

Providing a brief recruitment flow diagram or paragraph would enhance transparency.

Response: The manuscript now clearly describes how participants were recruited, including the use of facility gatekeepers, direct invitations, and referrals for healthcare professionals, family members, organizational leaders, and policymakers. We also added information on the number of individuals invited versus enrolled across study components, along with known reasons for non-participation, primarily related to scheduling and workload constraints. In addition, we clarified our saturation criterion, defining saturation as the point at which no new themes or substantive insights emerged from successive interviews or focus group discussions, and specified when saturation was reached for FGDs, IDIs, and KIIs. A brief recruitment flow paragraph has been included to enhance transparency. We appreciate the reviewer's suggestion, which has strengthened the methodological rigor and clarity of the manuscript.

The recruitment methods have been outlined on page 2, lines 50 – 54 to page 3, lines 1 - 19

10. Strengthen description of analytic procedures

To meet reproducibility standards, please expand on:

• Who conducted coding, their training, and whether coding was double-checked.

• How discrepancies were resolved.

• Which software was used (e.g., NVivo, ATLAS.ti) or manual coding.

Response: We have expanded the Data Analysis section to clearly describe who conducted the coding, their training, and the procedures used to enhance analytic rigor. We now state that coding was primarily conducted by the lead researcher, who has formal training in qualitative research methods and thematic analysis, as well as professional certification in group fitness instruction and licensure as a Zumba instructor, providing relevant contextual familiarity with physical activity behaviors. To enhance analytic rigor and credibility, supervisors with expertise in qualitative research provided expert review of the coding framework, theme development, and interpretive coherence of the findings. The analytic process was strengthened through iterative expert review and peer debriefing. Supervisors examined the alignment between the coded data, emerging themes, and interpretations, and provided structured feedback. Any discrepancies or areas of concern identified during this review were discussed and resolved through analytic refinement, including clarification of code definitions, consolidation or separation of themes where necessary, and re-examination of supporting data extracts.

Page 5, Lines 28 – 32

11. Data availability requirements
PLOS ONE requires underlying data to be publicly available unless restricted for ethical reasons. The current "available upon request" statement is insufficient.
Please either:
(a) deposit de-identified transcripts or coded datasets in a recognized repository (OSF, Figshare, institutional repository), providing DOI/accession numbers, or
(b) provide a justification for restricted access and describe the exact mechanism for data requests.

Response: This has been addressed above.

12. Reflexivity and researcher positionality
Please clarify:
• Who conducted the FGDs/IDIs/KIIs and their relationship to participants.
• Languages used, whether translators were involved, and how translation/back-translation quality was ensured.
• Whether member checking or participant validation was used.
This improves the manuscript's trustworthiness.

Response: We have revised the manuscript to provide a clearer and more comprehensive description of reflexivity and researcher positionality in line with COREQ guidelines. All focus group discussions (FGDs), in-depth interviews (IDIs), and key informant interviews (KIIs) were conducted by the lead researcher, a public health physician with formal training in qualitative research methods and thematic analysis. The researcher had no prior personal or professional relationships with participants, and no authority or supervisory roles existed between the researcher and participants at the time of data collection. This has now been explicitly stated in the manuscript. We have also clarified that data collection was conducted solely in English, and no interpreters were used.

Regarding participant validation, formal member checking was not conducted due to logistical constraints. This has been transparently acknowledged in the manuscript. We have detailed alternative strategies used to ensure rigor, including iterative reflexive analysis, peer debriefing, use of verbatim participant quotations to support themes, and maintenance of a detailed audit trail documenting analytic decisions.

Page 3, lines 53 - 56

13. Reporting guidelines
Please submit a completed COREQ checklist as supporting information to ensure full adherence to qualitative reporting standards.

Response: This has been done.

14. Strengthen data–claim alignment
The findings are relevant and coherent, but several policy recommendations (e.g., onsite gyms, scheduled work breaks, intersectoral urban-planning solutions) should be tied more explicitly to participants' quotes or existing evidence. Avoid generalizing beyond what qualitative data can assert.

Response: We have removed any over-generalisations and revised the manuscript to ensure that policy recommendations are clearly framed as participant-derived suggestions rather than definitive or universal prescriptions. Recommendations such as on-site gyms, scheduled work breaks, and intersectoral urban-planning approaches were explicitly raised by participants during the FGDs and KIIs, and this is now made clearer in the text.

To maintain brevity, not all illustrative quotations were included; however, the recommendations presented are grounded in the qualitative data and aligned with the identified themes. We have also adjusted the language throughout to avoid extending interpretations beyond what the qualitative evidence can support.

15. Minor edits
• Ensure consistent abbreviations and formatting across tables.
• Add identifiers to all participant quotations for traceability.
• Light proofreading for grammar and clarity.

Response: We have ensured consistent use of abbreviations and formatting across all tables and added identifiers to all participant quotations to enhance traceability. Regarding language, participant quotations have been intentionally left verbatim and not grammatically corrected, in keeping with qualitative research best practice, to preserve the authenticity and meaning of participants' voices. Minor grammatical and stylistic edits were applied only to the authors' narrative text to improve overall clarity and readability.

1. You need to state clearly how participants were recruited for the FGD, IDI, and KII.

Response: This has been addressed above. Page 2, Lines 50 – 54 to Page 3, Lines 1 - 4

2. You need to expand on the data collection process. How was the FGD, IDI, and KII conducted?

Response: We have expanded the Data Collection section to provide a more detailed description of how the FGDs, IDIs, and KIIs were conducted. Specifically, we have clarified the rationale for each method and development of each tool based on the Social Ecological Model, participant composition and setting for FGDs, procedures for moderating discussions and managing group dynamics, and the conduct of IDIs and KIIs, including selection of participants, interview settings, duration, and use of probes. We have also detailed the language of data collection, audio-recording procedures, use of field notes, informed consent, and the process for determining thematic saturation.

Page 3, Lines 30 – 51

3. How and when was saturation achieved?

Response: We have clarified the process for determining data saturation in the manuscript. Saturation was assessed concurrently with data collection and analysis through ongoing review of transcripts and field notes. After successive FGDs, IDIs, and KIIs, no new codes or substantive themes emerged, and existing themes were consistently reinforced across participant groups. Recruitment and data collection were discontinued at the point where additional interviews did not yield new insights, indicating that thematic saturation had been achieved. This clarification has been added to the Data Collection section of the manuscript.

Page 3, Lines 50 – 53

4. How many sessions of FGD, IDI and KII were held and why did you stop.

Response: We have clarified the number of FGDs, IDIs, and KIIs conducted in the revised manuscript. In total, 6 FGDs, 4 IDIs, and 5 KIIs were conducted. Data collection proceeded alongside concurrent analysis, and recruitment was discontinued once thematic saturation was achieved. Specifically, successive sessions no longer yielded new codes or substantive insights across the levels of the Social Ecological Model, indicating that additional data collection was unlikely to contribute further analytical depth. This information has now been added to the Data Collection section of the manuscript.

Page 3, Lines 4 - 18

5. How was data analysed? You need to expand on the analysis of the data

Response: The Data Analysis section has been expanded to provide a more detailed description of the analytic process. Specifically, we have clarified that data were analysed using a thematic analysis approach guided by the Social Ecological Model, incorporating both deductive and inductive coding. We have described the development of the initial coding framework, the iterative process of coding and theme refinement, and the use of both software-assisted (Dovetail) and manual analytic techniques, including memo writing, code mapping, and theme clustering. We have also detailed the involvement of multiple coders. These revisions have been incorporated into the Data Analysis section of the manuscript.

Page 3, Lines 5 - 25
